# European guidelines on radiographic image quality in chiropractic practice – proposal of a cross-sectional graded classification reporting principle

**DOI:** 10.1186/s12998-021-00375-4

**Published:** 2021-05-26

**Authors:** Klaus Doktor, Maria Lind Vilholm, Aldis Hardardóttir, Henrik Wulff Christensen, Jens Lauritsen

**Affiliations:** 1 Research Unit for Clinical Biomechanics, University of Southern Denmark, Campusvej 55, 5250 Odense M, Denmark; 2 Private Chiropractic Practice, Back Center Midwestern Jutland, Dalgas Allé 2A, 7400 Herning, Denmark; 3 Nordic Institute of Chiropractic and Clinical Biomechanics, Campusvej 55, 5230 Odense M, Denmark; 4 Private Chiropractic Practice, Reykjavik, Iceland; 5 Institute of Clinical Medicine, University of Southern Denmark and Orthopedic Department, Odense University Hospital, Odense, Denmark

**Keywords:** Image quality, Quality assurance, Guidelines, Lumbar spine, Radiograph, Primary care, Radiation dose

## Abstract

**Background:**

The Commission of the European Communities (CEC) has published: European Guidelines on Quality Criteria for Diagnostic Radiographic Images. These guidelines are considered a gold standard, recommended for use in quality assurance protocols.

The objectives of this study: 1) Propose a graded classification format for Danish chiropractic clinics according to the CEC-quality criteria for diagnostic radiographic images of the lumbar spine. 2) Propose a reporting principle for quality of radiographic images. 3) Document variation in radiation exposure among clinics.

**Methods:**

This is a cross-sectional study of image quality based on random sampling from 148 chiropractic clinics. Clinics were included if using: 1) Digital radiography and 2) The chiropractic picture and archiving system (KirPACS) at the Nordic Institute of Chiropractic and Clinical Biomechanics (NIKKB) in Denmark. A sample of 296 lumbar spine series were randomly collected from KirPACS (January 2018). Two independent observers reviewed 50 lumbar spine series twice with a 4-week interval, testing intra- and inter-observer reproducibility. The same observers then reviewed the remaining 246 radiographic studies. All studies were evaluated using the CEC Quality Criteria. Patient radiation dose values were retrieved from KirPACS (First quarter of 2020).

**Results:**

A reporting and classification principle of diagnostic image quality was used in 148 chiropractic clinics. Compliance with the 22 CEC Quality Criteria had proportions ranging from 0.72–0.96 for 18 criteria, while 4 criteria specifying detail and definition ranged between 0.20–0.66. The proposed rating system (A to E) revealed: 18 A clinics, 28 B clinics, 32 C clinics, 25 D clinics and 45 E clinics (A = highest quality; E = lowest quality). The patient radiation reference dose in Denmark is 7 mGy for the AP/PA lumbar spine. Very few clinics exceed the reference dose value, approximately 50% of clinics were below 5 mGy.

**Conclusion:**

A reporting principle is proposed for a graded classification format based on the CEC-quality criteria for diagnostic radiographic images of the lumbar spine. The Quality Criteria are for the most part met satisfactorily in 148 Danish chiropractic clinics, but important image details are compromised, in most cases, because of low patient radiation doses. The results of a patient radiation dose survey enabled documentation of variation in radiation exposure among chiropractic clinics.

**Supplementary Information:**

The online version contains supplementary material available at 10.1186/s12998-021-00375-4.

## Background

In “The Commission of the European Communities (CEC) trial on quality criteria for diagnostic radiographic images: Detailed results and findings”, the following is stated in the preamble:

“Quality and Safety have become hallmarks for efficient and successful medical intervention. A comprehensive quality and safety culture including mechanisms for audit has been progressively developed through the European Union with regards to the medical use of ionizing radiation. This concept has been integrated into various branches for diagnosis and treatment [1].”

At the same time, a protocol for quality assessment of lumbar spine radiographs is proposed by the CEC in: “European Guidelines on Quality Criteria for Diagnostic Radiographic Images [2].” These guidelines are the results of a European cooperation between the various professionals and authorities involved in diagnostic radiology. They have been revised over the years but continue to be the gold standard for image quality and quality assessment of radiographic images (see Additional file 1: A and B for details) [2].

In Denmark, chiropractors were authorized by the Danish National Board of Health in 1992 and have a 5-year Master’s program in Clinical Biomechanics from the University of Southern Denmark, where they receive an extensive education in diagnostic radiology among many other diagnostic and examination skills [3]. Chiropractors can, aside from radiography, draw from various other imaging modalities including musculoskeletal ultrasound and CT/MRI. To receive authorization an additional 1-year post-graduate internship in clinical practice is required. As seen generally in the Danish Health Care system for other health care providers, radiography is the most frequently used imaging modality and continues to also play an important role for chiropractors in diagnosis and patient management [4].

Primary chiropractic practice (2016) is organized in 249 clinics throughout the 5 regions in Denmark; about 200 with their own radiographic imaging systems [5]. Almost all (178 of about 200) clinics with their own radiographic systems are organized under supervision from the Diagnostic Imaging Unit at the Nordic Institute of Chiropractic and Clinical Biomechanics, where a physicist in charge assures that individual clinics meet the Danish Regulations on the use of Ionizing Radiation [6].

In 2008 a national Picture Archiving and Communication System (KirPACS) was established for chiropractic clinics using digital imaging systems. Today KirPACS has evolved to become a cornerstone in quality assurance systems for imaging and radiation dose monitoring and all technical service information and various system test results are also archived in the system.

### Optimization of radiation dose and image quality is an important part of the quality assurance procedures and must be performed with no more than two-year intervals

In this study, a cross-sectional evaluation of all clinics is reported, using the comprehensive evaluation protocols proposed by the European Commission, as part of the mandatory quality assurance program at NIKKB and compared to average radiation dose calculations based on data from 148 out of 170 clinics with digitized radiography systems and subscribing to supervision by NIKKB’s responsible physicist under the Danish National Board of Health’s Radiation Protection Program. At the time of the data collection, for various technical reasons, some clinics were still in the process of establishing a connection to KirPACS, explaining why 22 clinics are not included in this study.

The use of the European Quality Criteria have been found to be a reliable method of measuring image quality and have been used for almost two decades among Danish chiropractors. A previous report of a pilot study concluded that this method was suitable and recommended for radiographic image quality assurance programs within the Danish chiropractic profession [7]. This paper is following a recent publication on the reproducibility of the use of the CEC Quality Criteria [8].

### Objectives

The objectives of this study were to:

1. Propose a reporting principle for individual clinics in relation to quality of radiographic imaging.
2. Propose a graded classification format based on the CEC-quality criteria for diagnostic radiographic images of the lumbar spine.
3. Document variation in radiation exposure among chiropractic clinics in Denmark.

### Design

This is a double-blinded cross-sectional study of radiographic image quality based on random sampling.

## Materials and methods

### Data collection

The inter- and intra-observer reproducibility study included 50 radiographic studies of the lumbar spine and has been reported in full detail in a separate paper [8].

The assessment of the diagnostic quality of lumbar spine radiographs in chiropractic practice in Denmark, included clinics: 1) Using digitalized radiographic imaging systems and, 2) Storing studies in KirPACS at NIKKB in Odense, Denmark. The study was initiated in January 2018 and was completed in the first quarter of 2020.

The project was initiated by anonymizing and numbering all studies. The study reviewers or clinicians obtaining the radiographs were blinded to the identity of patients/clinics and potential participation in the quality assurance procedure. After randomly retrieving 2 studies per clinic from 148 clinics in KirPACS, the studies were analyzed using the image viewer Osirix version 5.7.1.for Mac and a digitized format of the CEC: Quality Criteria for Diagnostic Radiographic Images. The results were tabulated directly into a software module made in Epidata Entry Client and Epidata Manager (version 2.0.7.22r547) [9]. Acceptance tested, high resolution (2 million pixel) diagnostic monitors (BARCO MDNC-2121) were used for the image evaluation process [10]. Two observers, licensed chiropractors, with 2 years of clinical experience handled the readings of all the samples. The two observers were blinded to patient and diagnostic information and did not have access to previous readings, and efforts were made to minimize confounding factors, such as visible clinic identification or modality manufacture information, during the readings. The observers were given 4 weeks to finish their evaluations and could log on and off to access the images any time they wished. After the initial evaluations of 50 studies for the reproducibility study (reported separately), the remaining studies were divided in two portions, one for each observer. Observers could evaluate in any order they wished within the timeframe.

### Statistics and proposed quality definition groups

All analysis was performed using STATA 15 for Windows, Stata Corporation, USA [11] and Microsoft Excel 2010, Microsoft Office Package, Microsoft Corporation, USA [12]. Statistical analysis was carried out at either measurement level, clinic level or lumbar projection level (AP/PA; L1-L4; L5/S1) as indicated in the tables and graphs.

Quality in this study was defined according to the CEC Quality Criteria as: Ok = Sum of correct (acceptable) measurements; error = Sum of incorrect (not acceptable) measurements; diff. = Difference between measurements within a given clinic (variation).

All clinics were ranked according to counts of: “error” (sum of not acceptable in both measurements), “Ok” (count of correct in both measurements) and “stability” (number of differences in assessment of the two measurements) and classified in 5 percentile groups (A: 0–10, B: 11–40, C: 41–59; D: 60–89, E: 90+) with poor quality as (errors = highest; ok = lowest; diff = highest) for each projection.

All clinics were then classified on the combination of the percentile groups for all projections and measurements, as follows: Overall A: (only A grading), B: (B or A grading), C: (No D grading), D: (maximum one E grading), E: (two or more E). Since probit plots showed that totals for ok, error and diff were all reasonably Gaussian distributed we also applied an alternative according to (A: < mean-2 SD; B < mean-1 SD; C: mean +/− 1 SD; D: > mean + 1 SD; E: >mean + 2 SD) based on I Chart graph values (EpiData Analysis, www.epidata.dk) for all measurements.

## Results

These are the results of a study of radiographic image quality based on “European Guidelines on Quality Criteria for Diagnostic Radiographic Images” (EUR 16260). The study was performed as part of the quality assurance program for 148 chiropractic clinics in Denmark using computerized radiography or direct radiography (CR or DR systems) in their primary care practice. A total of 296 lumbar spine studies were retrieved from KirPACS, analyzed and scored according to the proposed image criteria.

### Compliance with the CEC diagnostic image quality criteria

In Table 1, the global results for 148 Danish chiropractic clinics is presented for Lumbar Spine projections (AP/PA, Lateral L1-L4 and Lateral L5/S1), as a percentage of the total sample size fulfilling the individual CEC Diagnostic Quality Criteria in this study.

**Table 1:** Global compliance in Danish chiropractic clinics to the individual CEC Quality Criteria for Diagnostic Radiographic Images – all measurements presented

Quality Criteria	Proportion correct (*n* = 296)	95% confidence interval
**Lumbar Spine AP/PA projection**		
1.1.1. Visually sharp reproduction of the upper and lower-plate surfaces	0.89	(0.85–0.92)
1.1.2. Visually sharp reproduction of pedicles	0.93	(0.89–0.95)
1.1.3. Reproduction of the intervertebral joints	0.81	(0.76–0.86)
1.1.4. Reproduction of the spinous and transverse processes	0.84	(0.80–0.88)
1.1.5. Visually sharp reproduction of the cortex and trabecular structures	0.66	(0.60–0.71)
1.1.6. Reproduction of the adjacent soft tissues, particularly the psoas shadows	0.97	(0.94–0.98)
1.1.7. Reproduction of the sacro-iliac joints	0.90	(0.86–0.93)
1.2.1. Visually details down to diameter 0.3–0.5 mm	0.92	(0.88–0.94)
1.3.1. Image Acceptability (acceptable = scores 2 or 3)	0.72	(0.66–0.77)
**Lumbar Spine Lateral (L1-L4) projection**		
2.1.1. Visually sharp reproduction of the upper and lower-plate surfaces	0.92	(0.88–0.95)
2.1.2. Full superimposition of the posterior vertebral edges	0.82	(0.78–0.87)
2.1.3. Reproduction of the pedicles and the intervertebral foramina	0.96	(0.93–0.98)
2.1.4. Visualization of the spinous processes	0.79	(0.74–0.84)
2.1.5. Visually sharp reproduction of the cortex and trabecular structures	0.20	(0.15–0.25)
2.2.1. Visually details down to diameter 0.5 mm at 3rd lumbar vertebral body	0.37	(0.32–0.43)
2.3.1. Image Acceptability (acceptable = scores 2 or 3)	0.66	(0.60–0.71)
**Lumbar Spine Lateral (L5-S1)**		
3.1.1. Reproduction by tangential production of the inferior end plate of L5^a^	0.81	(0.76–0.86)
3.1.2. Visualization of the spinous process of L5	0.85	(0.80–0.89)
3.1.3. Visualization of the anterior border of the upper sacrum	0.96	(0.93–0.98)
3.1.4. Reproduction of the vertebral pieces of the upper sacrum	0.75	(0.70–0.80)
3.2.1. Linear and reticular details down to diameter 0.5 mm.	0.27	(0.22–0.32)
3.3.1. Image Acceptability (acceptable = scores 2 or 3)	0.78	(0.73–0.83)

*N* = 296 for all assessments (two per clinic), except for ^a^ (for one clinic only 1 assessment)

### Proposed grading system for individual clinics

In Table 2 the results for individual clinics are presented as a percentage of the fulfilled CEC Diagnostic Quality Criteria for all projections and divided into individual projections: Lumbar AP/PA, Lateral L1-L4 and Lateral L5/S1 projections. This allows for a ranking of clinics by percentiles from maximum to minimum. Comparison of clinics is crucial in pinpointing potential problem areas regarding the imaging quality. The maximal achievable scores for all projections are 44 points (AP/PA: 9 variables, Lateral L1-L4: 7 variables and, Lateral L5/S1: 6 variables = 22 variables total per series). The combined score for both series is: All projections 22 × 2 = 44 (AP/PA = 18, Lateral L1-L4 = 14 and Lateral L5/S1 = 12).

**Table 2:** Sum scores divided in: correct (compliance with quality criteria), errors (no compliance with quality criteria) and stability (same quality in both radiographic series per clinic) in assessments for all clinics. Mean (95% CI) and presented in percentile (p) groups with a proposed grading: A-E

	Mean	95% conf. interval	Min	p10	p25	Median	p75	p90	Max
**Correct**^**d**^ Proposed scoring^a^				**E**	**D**	**C**	**B**	**A**	
All projections	33.56	(32.69 34.43)	14	25	31	34	37	40	43
AP/PA	15.26	(14.88 15.65)	7	12	14	16	17	18	18
L1-L4	9.45	(9.11 9.78)	3	7	8	9	11	12	14
L5/S1	8.85	(8.50 9.21)	2	6	8	9	10	12	12
**Error**^**c**^ Proposed scoring^a^				**A**	**B**	**C**	**D**	**E**	
All projections	10.42	(9.55 11.29)	1	4	7	10	13	19	30
AP/PA	2.72	(2.34 3.10)	0	0	1	2	4	6	11
L1-L4	4.55	(4.22 4.89)	0	2	3	5	6	7	11
L5/S1	3.14	(2.79 3.50)	0	0	2	3	4	6	10
**Stability**^**b**^ Proposed scoring^a^				**E**	**D**	**C**	**B**	**A**	
All projections	5.20	(4.72 5.68)	0	2	3	5	7	9	14
AP/PA	1.96	(1.70 2.21)	0	0	1	2	3	4	6
L1-L4	1.76	(1.56 1.95)	0	0	1	2	2	3	5
L5/S1	1.49	(1.28 1.69)	0	0	0	1	2	3	5

^a^Proposed cut point in grading A-E. Only used for the “all projections” group. A = highest grade

^b^Stability in quality measured as whether the two assessed radiographic series had the same scoring (ok versus error)

^c^Sum of errors in all assessments. ^d^ Sum of correct assessments. Classified in grades according to mean +/− SD criteria, see Materials and methods section

The proposed grading makes it possible to combine all grades and present a final ranking of clinics based on a 3-letter classification/grading system, as can be seen below in Table 3.

**Table 3:** Median and range of errors and correct for all assessments ranked according to overall classification of clinics

Grade^a^	Number of Clinics	OK assessments (out of possible 44)		Errors (out of possible 44)	
		median	range	median	range
AAA	7	43	(41–43)	1	(1–3)
AAB	5	40	(40–42)	4	(2–4)
AAC	6	41	(40–41)	3	(3–4)
BBA	5	37	(37–39)	7	(5–7)
BBB	2	38	(38–38)	6	(6–6)
BBC	10	38	(37–39)	6	(5–7)
BBD	9	38	(37–39)	6	(5–7)
BBE	2	37	(37–37)	7	(7–7)
CCB	10	36	(34–36)	8	(8–10)
CCC	9	35	(35–36)	9	(8–9)
CCD	10	34	(34–35)	10	(9–10)
CCE	3	34	(34–34)	10	(10–10)
DCE	1	33	(33–33)	10	(10–10)
DDC	6	32	(32–33)	12	(11–12)
DDD	10	33	(32–33)	12	(11–12)
DDE	8	33	(32–33)	11	(11–12)
EDD	3	31	(31–31)	13	(13–13)
EDE	7	31	(31–31)	13	(12–13)
EEB	1	24	(24–24)	20	(20–20)
EEC	2	28	(26–30)	16	(14–18)
EED	9	25	(20–29)	19	(15–24)
EEE	23	28	(14–30)	16	(14–30)

^a^Grade nomination: First letter = Grading according to Ok. Second letter = Grading according to error. Third letter = Grading according to difference between the two assessments. Grades are defined according to ranking of all clinics. OK: Best to have a high number of OK = Grade AAA, and worst to have a low number of OK = Grade EEE. A: top 10% of the entire group; B: 11–25%; C: 26–50%; D: 51–75%; E: bottom 76–100%. Error + differences: Best to have a low number of Errors and/or Differences between x-ray series (homogenizes). Scale now reversed. Grade A: bottom 10% etc.

### Proposed reporting principle for individual clinics in relation to image quality in overview

**Fig. 1:**
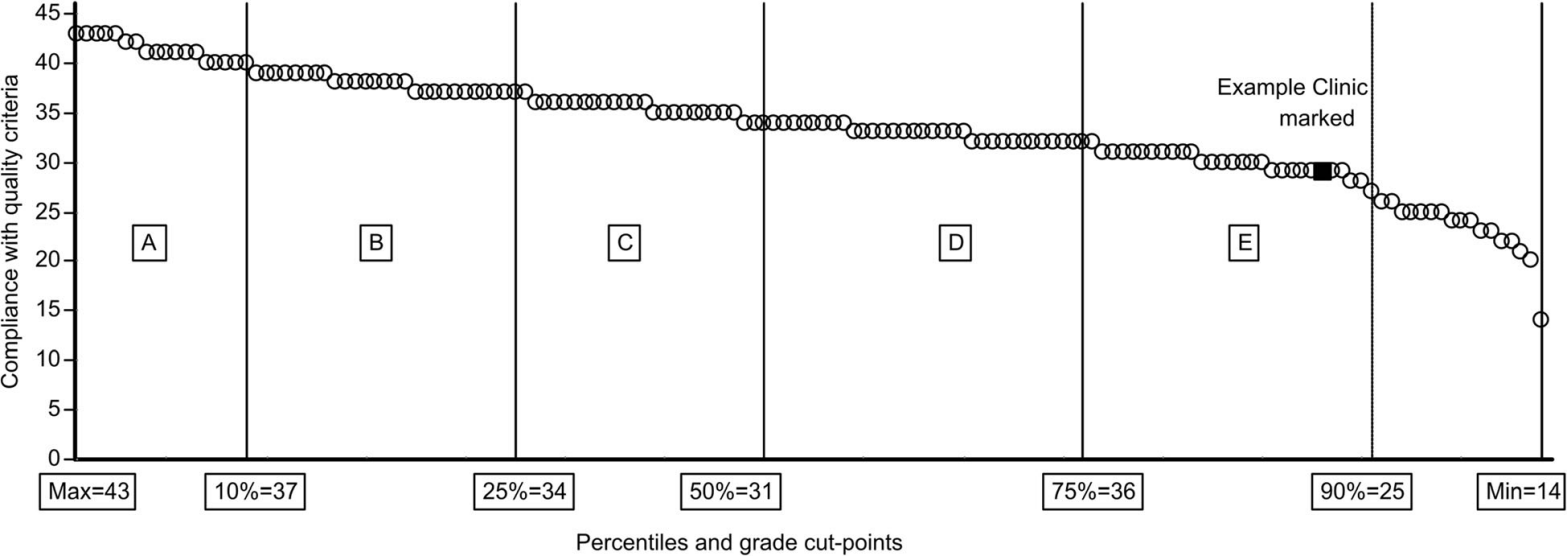
Compliance with the CEC Radiographic Quality Criteria for the lumbar spine by clinic with ranking and classification by number of correct assessments (Max. possible score = 44)

One of our objectives was to propose a reporting principle for individual clinics to present individual results in a simple and clear format, making it possible to compare the results with the rest of the group, as can be seen in Fig. 1 below.

### Variation in radiation exposure among clinics with electronic image storage (KirPACS)

Our last objective was to document the variation in radiation exposure among clinics connected to the KirPACS. Based on reporting to the responsible physicist, radiation exposure was calculated and is presented below in Fig. 4 and divided in 3 patient weight classes (below 51 kg, 51-89 kg and 90 kg and over). The reference dose level for patients 51–90 kg for chiropractors in Denmark is 7.00 mGy (entrance dose).

### Proposed ranking of individual clinics by patient radiation exposure

It is important to monitor clinic performance in relation to radiation exposure. In Fig. 5, the results of dose calculations are presented with a ranking of all clinics (based on 10 patient measurements per clinic), with indication of maximum, minimum and average doses for the sample clinic. Clinics to the left have lower patient doses and clinics towards the right have higher patient doses. All clinics receive this figure, where their own results are highlighted for comparison with other clinics (see sample clinic).

## Discussion

Global compliance in 148 Danish chiropractic clinics to the CEC Quality Criteria for Diagnostic Radiographic Images is presented in Table 1, indicating the proportion of all clinics meeting the individual quality criteria for lumbar radiographs. Overall, most of the quality criteria are met by clinic proportions of 80–90%. The AP/PA lumbar spine projections presented with high/low compliance rates from 97% (*criteria 1.1.6*) to 66% (*criteria 1.1.5*). The Lateral lumbar spine L1-L4 projections from 96% (*criteria 2.1.3*) to 20% (*criteria 2.1.5*) and 37% (*criteria 2.2.1*). The Lateral L5/S1 projection ranged from 96% (*criteria 3.1.3*) to 27% (*criteria 3.2.1*). To identify the causes for the low proportion of clinics meeting: *Criteria 2.1.5 (Visually sharp reproduction of the cortex and trabecular structures); Criteria 2.2.1 (Visually details down to 0.5 mm at 3rd lumbar vertebral body, ventral edge), and criteria 3.2.1 (Linear and reticular details down to 0.5 mm in width),* it is necessary to analyze the characteristics of these three criteria. They all require the highest levels of visibility of details, as described in the CEC-document and it is evident that the image quality is indeed compromised for the two lateral lumbar spine projections. The two latter criteria 2.2.1. and 3.2.1. are absolute measurements of details in millimetres and only 37 and 27% of the images fulfilled these. Criteria 2.1.5. was only met in 20% of the images. This is a problem since the common trait for these three criteria is an objective threshold for measurable detail in order to meet the diagnostic needs. Typically, about 90% of the image quality criteria are fulfilled by radiographs regarded as acceptable for clinical diagnosis and when there is a trade-off between patient dose and image quality, it is necessary for both quantities to be measured [13]. In Denmark, the EU-regulations on diagnostic use of radiation have caused a one-sided focus on patient radiation exposure, without enough attention to radiographic image quality and generally with deteriorating effects on the radiographic image quality throughout the healthcare system. The problem is built into the way reference doses are determined by the health authorities. According to the CEC publication, the criteria for radiation dose to the patient are expressed in terms of a reference dose level, based on the third quartile (75th percentiles) values as seen in earlier European patient dose surveys. Its purpose, if exceeded, is to: “Initiate an immediate investigation into the reasons for using relatively high dose techniques and to trigger appropriate corrective action. The reference dose value can be taken as an upper limit from which progress should be pursued to lower dose levels in line with the ALARA (as low as reasonably achievable) principle”. By this method the (earlier) recommended Entrance Surface Dose for an average sized patient (assumed to be 20 cm AP diameter and 70 ± 3 kg) was derived at 10 mGy for AP/PA, 30 mGy for lateral lumbar spine and 40 mGy for lateral lumbosacral spine. Paradoxically, the administration of the crucial dose/quality balance, has caused more problems for workplaces and professions, the reason being that new and lower dose reference values are implemented by authorities after the third quartile principle. In other words, when chiropractic clinics (and hospitals), because of dose/image optimization, have become more and more homogeneous, the reference dose has repeatedly been lowered, thus pushing the radiation doses below the point of acceptable balance between dose and quality. At the time when image evaluations in this study were performed, the reference dose had already been lowered from 10 to 7 mGy for an AP/PA lumbar spine [6, 14, 15]. Today, the value is close to half of the initially recommended dose of 10 mGy [16]. This was never the intention as described in CEC-publication EUR 16260 [2] and in ICRP 90 and 103, and adding the fact that there has been a transition from film-based to less sensitive digital radiography, this has further challenged the diagnostic image quality. Methods for measurement of patient dose are comparatively well established. Assessment of image quality is less straight forward. Image quality is affected by resolution, sensitivity and statistical noise [13].

In Table 2, results are shown at projection level. We pooled all projections and ranked according to scores producing a classification system, specifying the association between grades (A-E) and scoring, based on the achieved scores for all three projections combined (max. 44 points possible) and then divided into projections and classified in percentiles (p10, p25, p50, p75 and p90). This allows us to compare projections and to have a general view of projections in relation to “OK”, “error” and “stability”, i.e. all projections are shown to have a mean score of 33.56 correct assessments of 44 possible, with a minimum score of 14 and a maximum score of 43. As can be seen the 90-percentile threshold for the grade A is equivalent to a minimum score of 40 correct.

In Table 3, results are classified at clinic level. It can be derived how the grade A-group of clinics, B-group etc., are defined. In this table the division of 148 clinics into group A, B, C, D and E is specified.

**Fig. 2:**
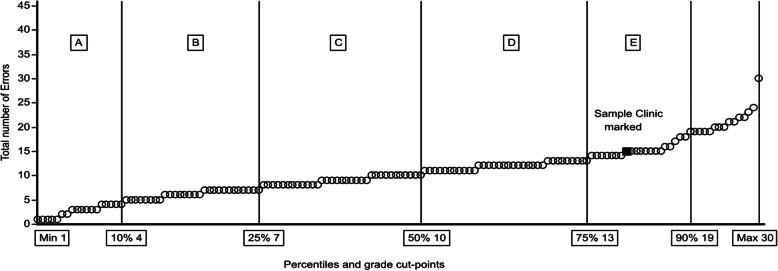
Non-compliance with the CEC Radiographic Quality Criteria for the lumbar spine by clinic with ranking and grouping by number of incorrect (error) assessments. Percentiles and grade cut-points

**Fig. 3:**
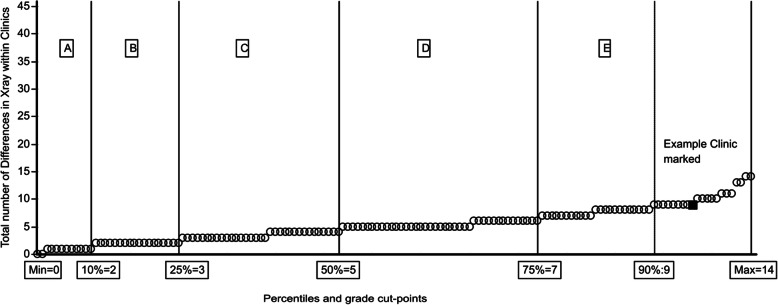
Stability of lumbar spine Radiographic Quality by clinic with ranking and grouping by number of differences in assessment. Percentiles and grade cut-points

In Figs. 1, 2 and 3, part of the proposed feedback and reporting system to clinics is presented, with all clinics ranked and classified. Clinics are represented as small circles and in this example with a blue indication of the performance of an individual clinic. This type of presentation makes it very easy for clinics to see their results and compare to others.

**Fig. 4:**
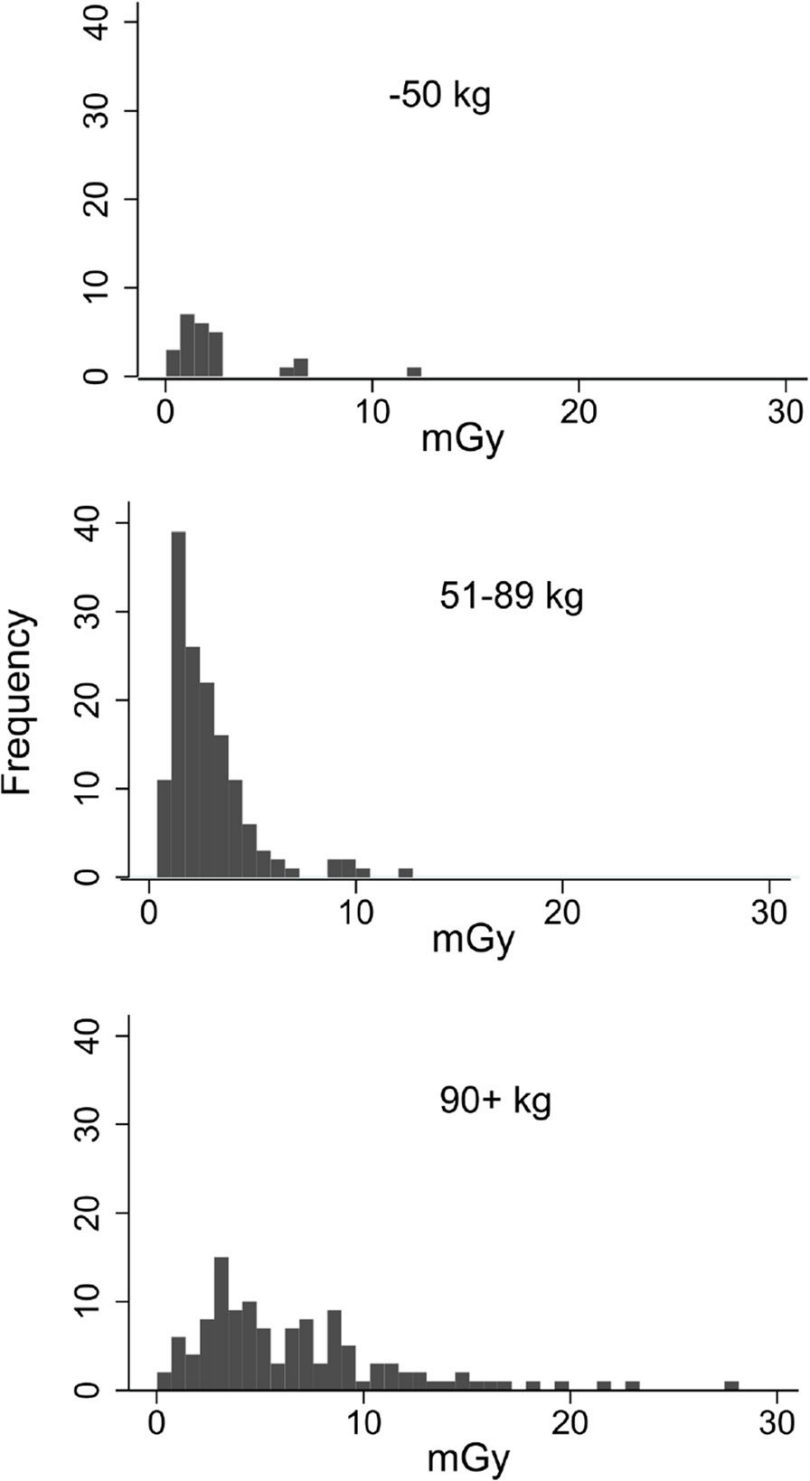
Distribution of patient entrance radiation dose (mGy) by weight classes

Figure 4 displays the distribution of patient radiation dose by patient weight-classes. The reference dose level for patients 51–89 kg is 7.00 mGy, and as can be seen most values are clearly below this value. As mentioned earlier in the Discussion section, the radiation dose has, in many places, been reduced to a critical level, sacrificing adequate image quality. The mean patient dose value is 4.5 mGy for patients in this weight group.

**Fig. 5:**
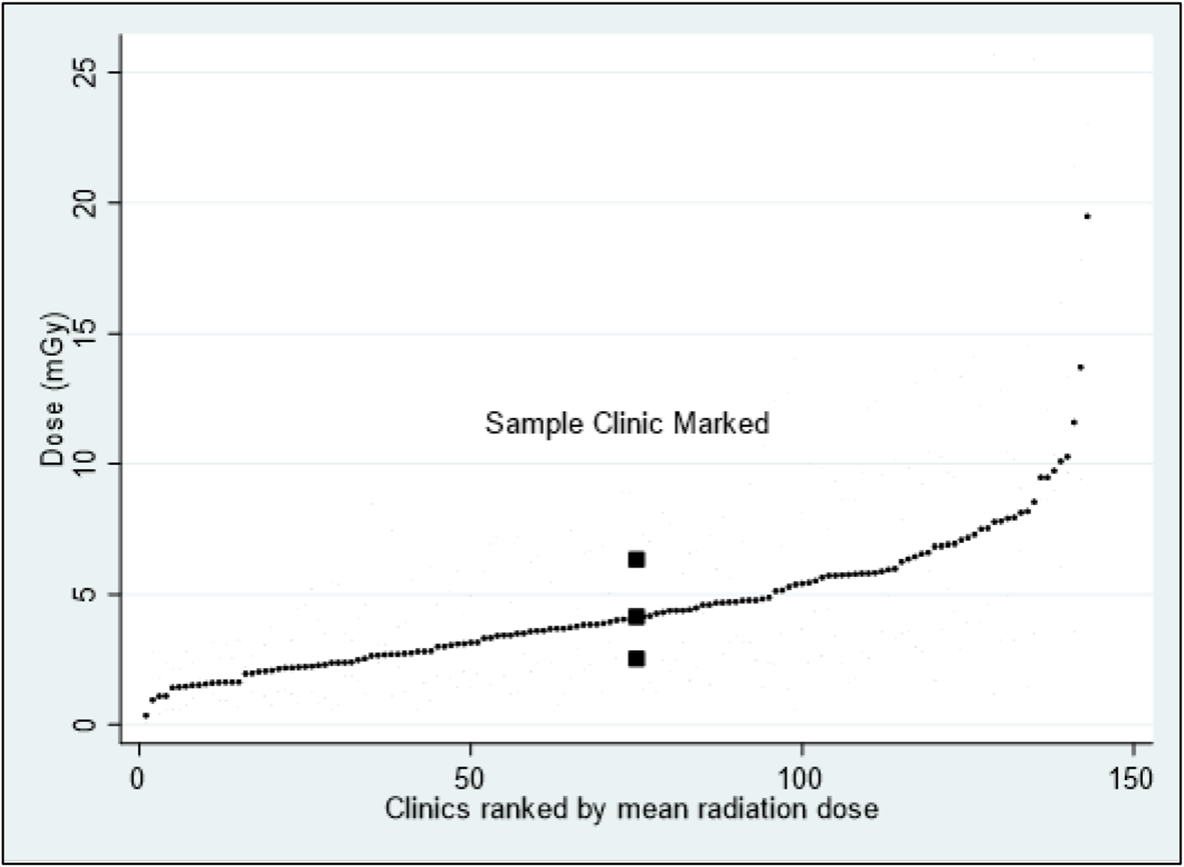
Clinics ranked by radiation dose. Min./max. and mean doses will be displayed in reports for each clinic individually, as seen highlighted for a sample clinic

Figure 5 presents clinics ranked by radiation dose and with min./max. and mean doses for an individual clinic indicated. There is a clear impression of patient dose values being quite low. For this study, entrance doses have been calculated based on patient data (weight, height, distance, kV and mAs).

It is important to be aware of the limitations in this study, primarily the section reporting the radiation dose calculations. The radiation doses presented are the result of exposure data collected on 10 patient examinations of the lumbar spine in all clinics in this study and are not necessarily representative of radiation doses used for the rated images in this trial. Therefore, no comparison of dose and image quality has been made at clinic level in this paper. Ideally doses at individual image level should be possible to report on, establishing a crucial foundation to build image quality assurance programs. It is currently being discussed how an automated dose management system can be implemented among Danish chiropractors. To our knowledge, this will be the first place in the world, where a chiropractic profession introduces automated radiation dose passports for patient safety. This will also allow an introduction of more precise and systematic dose area product (DAP) measurements at projection level. Another limitation could potentially be the use of relatively inexperienced observers, although producing acceptable inter- and intrarater agreement coefficients. This, on the other hand, indicates that the CEC Quality Criteria can be used reliably among clinicians as routine quality assurance measures.

## Conclusion

1. We are proposing a reporting principle for individual chiropractic clinics in Denmark, in relation to the quality of lumbar spine radiographs produced. This quality reporting system will be recommended for future quality assurance programs carried out at 2-year intervals. Although these criteria can be helpful for clinicians in daily practise, the reporting system is intended for large scale national studies as part of quality assurance programs.
2. The CEC-Quality Criteria are, for the most part, met satisfactorily in 148 Danish chiropractic clinics, but important image details are generally compromised for the lateral lumbar spine projections, in most cases, because of low patient radiation doses. This is not acceptable and needs attention.
3. It is also proposed that a graded classification format based on the CEC-quality criteria for diagnostic radiographic images of the lumbar spine is implemented at national level. This classification scheme can be carried out at clinic level and at image projection level. It is recommended that resources, also internationally, are allocated to implement the proposed scheme or similar.
4. The results of a patient radiation dose survey have enabled a documentation of variations in radiation exposures among chiropractic clinics with electronic image storage (KirPACS). The new EU-regulative on the use of ionizing radiation for diagnostic imaging is in effect and necessitates DAP-meters to be installed on all radiographic installations in chiropractic practice. It is recommended that resources are raised for this implementation and for a central administered dose monitoring system.
5. A quality system could be implemented globally to ensure a high standard of radiographs produced in chiropractic clinics.

## Supplementary Information

**Additional file 1.**

## Data Availability

The datasets used and/or analyzed during the current study are available from the corresponding author on reasonable request.

## Abbreviations

CEC: Commission of the European Communities
PACS: Picture Archiving and Communication System
NIKKB: Nordic Institute for Chiropractic and Clinical Biomechanics
KirPACS: Chiropractic Picture Archiving and Communication System
CR: Computed radiography
DR: Direct radiography
AP: From anterior to posterior
PA: From posterior to anterior
L1: First lumbar vertebra
L4: Fourth lumbar vertebra
L5: Fifth lumbar vertebra
S1: First sacral vertebra
L/S: Lumbo-sacral junction
DAP: Dose area product
