# Supplementary material for: European guidelines on radiographic image quality in chiropractic practice – proposal of a cross-sectional graded classification reporting principle
Source: Chiropr Man Therap. 2021 May 26;29:19. doi: 10.1186/s12998-021-00375-4 (PMC8152339; doi:10.1186/s12998-021-00375-4)
Supplement: Supplementary file 1 — Additional file 1. [file 12998_2021_375_MOESM1_ESM.docx]

## *Additional files (Elements from Quality Assurance Handbook of Radiography, Chiropractic Knowledge Hub, University of Southern Denmark).*

***A: Variables for EU-image criteria, important details and general assessment***

1. **LUMBAR SPINE AP/PA Projection**
   1. **Image Quality Assessment Criteria**
      1. Visually sharp reproduction, as a single line, of the upper and lower-plate surfaces in the centered beam area.
      2. Visually sharp reproduction of the pedicles.
      3. Reproduction of the intervertebral joints.
      4. Reproduction of the spinous and transverse processes.
      5. Visually sharp reproduction of the cortex and trabecular structures.
      6. Reproduction of the adjacent soft tissues, particularly the psoas shadows.
      7. Reproduction of the sacro-iliac joints.
   2. **Important Image Details**
      1. Visually details down to 0.3-0.5 mm.
   3. **General Assessment**
      1. Film acceptability.
2. **LUMBAR SPINE Lateral L1-L4 Projection**
   1. **Image Quality Assessment Criteria**
      1. Visually sharp reproduction of the upper and lower-plate surfaces represented as lines with the resultant visualization of the intervertebral spaces.
      2. Full superimposition of the posterior vertebral edges.
      3. Reproduction of the pedicles and the intervertebral foramina.
      4. Visualization of the spinous processes.
      5. Visually sharp reproduction of the cortex and trabecular structures.
   2. **Important Image Details**
      1. Visually details down to 0.5 mm. at 3^rd^ lumbar vertebral body, ventral edge.
   3. **General Assessment**
      1. Film acceptability.
3. **LUMBAR SPINE Lateral L5/S1 Projection**
   1. **Image Quality Assessment Criteria**
      1. Reproduction by tangential production of the inferior end plate of L5 and the superior end plate of S1.
      2. Visualization of the spinous process of L5.
      3. Visualization of the anterior border of the upper sacrum.
      4. Reproduction of the vertebral pieces of the upper sacrum.
   2. **Important Image Details**
      1. Linear and reticular details down to 0.5 mm. in width.
   3. **General Assessment**
      1. Film acceptability.

***B: Variable-definitions of the EU-image criteria***

1. **LUMBAR SPINE AP/PA Projection**
   1. **Image Quality Assessment Criteria ***
      1. Visually sharp reproduction of the upper and lower-plate surfaces represented as lines in the centered beam area.
      2. Visually sharp reproduction of the pedicles.
      3. Reproduction of the intervertebral joints (apophyseal or facet).
      4. Reproduction of the spinous and transverse processes.
      5. Visually sharp reproduction of the cortex and trabecular structures.
      6. Reproduction of the adjacent soft tissues, particularly the psoas shadows.
      7. Reproduction of the sacro-iliac joints.
   2. **Important Image Details****
      1. Image details (3^rd^ lumbar vertebral body) down to 0.3-0.5 mm.
   3. **General Assessment *****
      1. Film acceptability.
   4. **Scoring (maximum 11 points)**
      1. ***Image Quality Assessment Criteria** Image criteria fulfilled = 1 Image criteria is not fulfilled = 0 If any area obscured by a pathological condition, then “P” should be placed in the appropriate box.
      2. **** Important Image Details** Image details fulfilled = 1 Image details not fulfilled = 0
      3. ***** General Image Acceptability** Fully acceptable = 3 Probably acceptable = 2 Only acceptable under limited clinical conditions = 1, give reasons (see below) Unacceptable = 0, give reasons (see below) **Noise**, defined as grainy, random fluctuations in the image which superimpose the image pattern (* = Optimal; - = Suboptimal; 0 = Unacceptable)  **Contrast**, defined as differences in optical density between object and background (* = Optimal; + = Too high; - = Too low) **Sharpness**, defined as the extend of the transition zone for optical density between object and background (* = Optimal; - = Sub-optimal; 0 = Unacceptable) **Collimation**, defined as x-ray beam limitation (* = Optimal; + = Field size too large; - = Field size too small) **Patient positioning**, defined as mal-positioning of the patient in relation to the x-ray field (* = Optimal; - = Sub-optimal; 0 = Unacceptable)


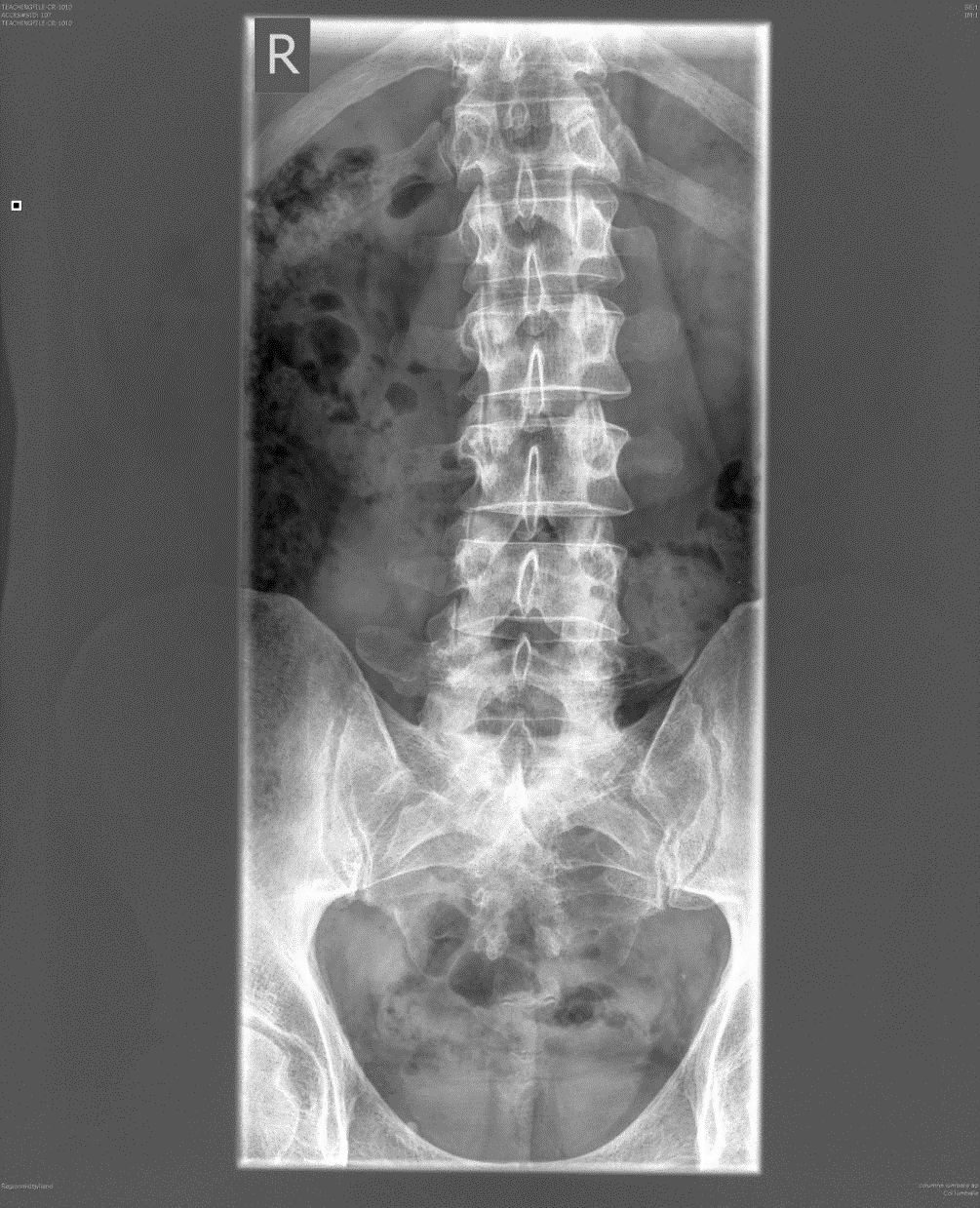


- - 1. **Psoas shadows**
    2. **Pedicles**
    3. **Endplates as a line in center**
    4. **Intervertebral joints**
    5. **Spinous or transverse processes**
    6. **Cortex and trabecular structures**
    7. **Sacroiliac joints**

1. **LUMBAR SPINE Lateral L1-L4 Projection**
   1. **Image Quality Assessment Criteria ***
      1. Visually sharp reproduction of the upper and lower-plate surfaces represented as lines with the resultant visualization of the intervertebral spaces.
      2. Full superimposition of the posterior vertebral edges.
      3. Reproduction of the pedicles and the intervertebral foramina.
      4. Visualization of the spinous processes.
      5. Visually sharp reproduction of the cortex and trabecular structures.
   2. **Important Image Details****
      1. Visually details down to 0.5 mm. at 3^rd^ lumbar vertebral body, ventral edge.
   3. **General Assessment *****
      1. Film acceptability.
   4. **Scoring (maximum 9 points)**
      1. ***Image Quality Assessment Criteria** Image criteria fulfilled = 1 Image criteria is not fulfilled = 0 If any area obscured by a pathological condition, then “P” should be placed in the appropriate box.
      2. **** Important Image Details** Image details fulfilled = 1 Image details not fulfilled = 0
      3. ***** General Image Acceptability** Fully acceptable = 3 Probably acceptable = 2 Only acceptable under limited clinical conditions = 1, give reasons (see below) Unacceptable = 0, give reasons (see below)  **Noise**, defined as grainy, random fluctuations in the image which superimpose the image pattern (* = Optimal; - = Suboptimal; 0 = Unacceptable)  **Contrast**, defined as differences in optical density between object and background (* = Optimal; + = Too high; - = Too low) **Sharpness**, defined as the extend of the transition zone for optical density between object and background (* = Optimal; - = Sub-optimal; 0 = Unacceptable) **Collimation**, defined as beam limitation (* = Optimal; + = Field size too large; - = Field size too small) **Patient positioning**, defined as mal-positioning of the patient in relation to the x-ray field (* = Optimal; - = Sub-optimal; 0 = Unacceptable)

**and**
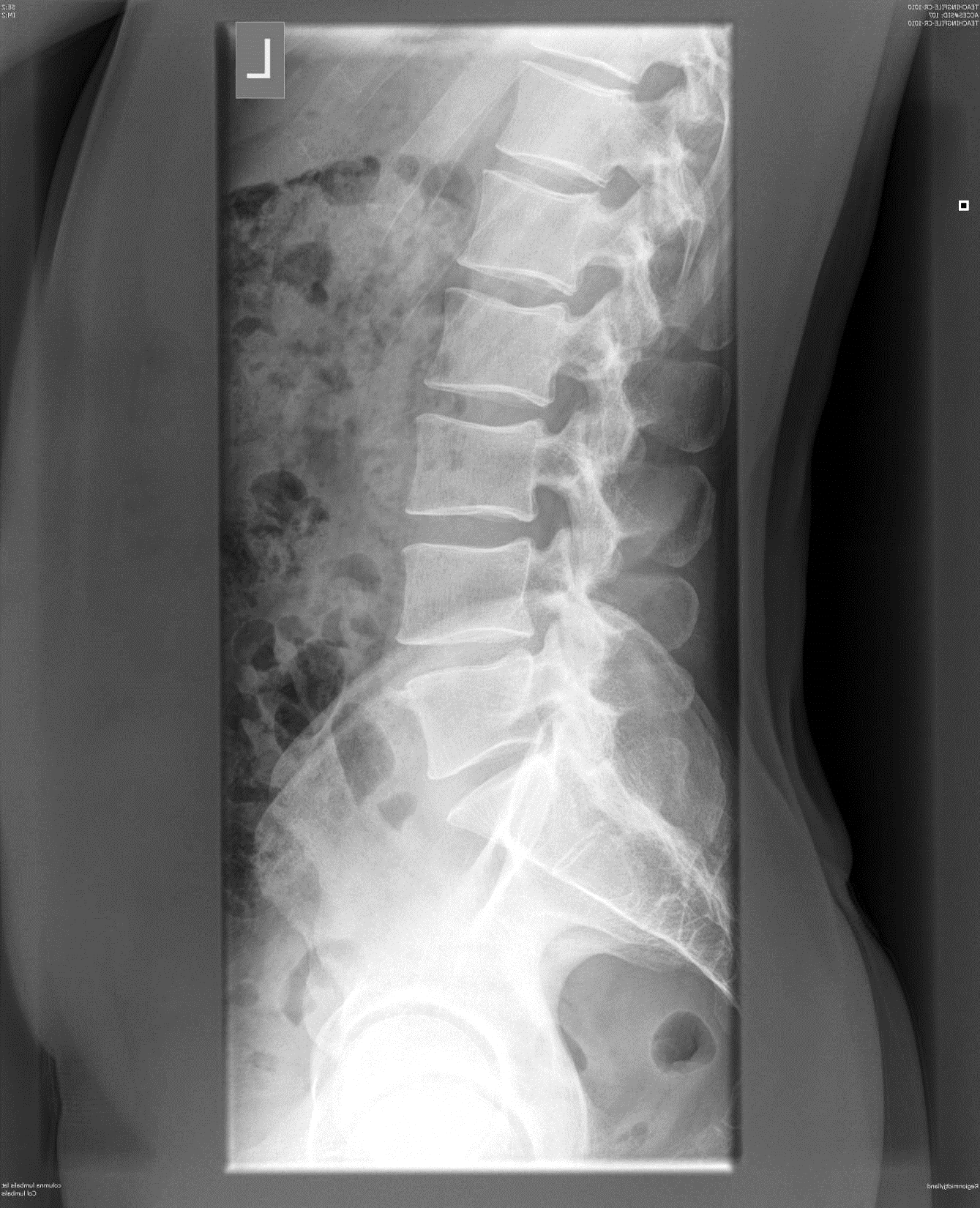


**2.1.5. Cortex and trabecular structures**

**2.1.4. Spinous proces**

**2.1.3. Pedicles and foramina**

**2.1.2. Superimposition of posterior vertebral edges**

**2.1.1. Endplates and intervertebral space**

1. **LUMBAR SPINE Lateral L5/S1 Projection**
   1. **Image Quality Assessment Criteria ***
      1. Reproduction by tangential production of the inferior end plate of L5 and the superior end plate of S1.
      2. Visualization of the spinous process of L5.
      3. Visualization of the anterior border of the upper sacrum.
      4. Reproduction of the vertebral pieces of the upper sacrum.
   2. **Important Image Details ****
      1. Linear and reticular details down to 0.5 mm. in width.
   3. **General Assessment *****
      1. Film acceptability.
   4. **Scoring (maximum 8 points)**
      1. ***Image Quality Assessment Criteria** Image criteria fulfilled = 1 Image criteria is not fulfilled = 0 If any area obscured by a pathological condition, then “P” should be placed in the appropriate box.
      2. **** Important Image Details** Image details fulfilled = 1 Image details not fulfilled = 0
      3. ***** General Image Acceptability** Fully acceptable = 3 Probably acceptable = 2 Only acceptable under limited clinical conditions = 1, give reasons (see below) Unacceptable = 0, give reasons (see below)  **Noise**, defined as grainy, random fluctuations in the image which superimpose the image pattern (* = Optimal; - = Suboptimal; 0 = Unacceptable)  **Contrast**, defined as differences in optical density between object and background (* = Optimal; + = Too high; - = Too low) **Sharpness**, defined as the extend of the transition zone for optical density between object and background (* = Optimal; - = Sub-optimal; 0 = Unacceptable) **Collimation**, defined as beam limitation (* = Optimal; + = Field size too large; - = Field size too small) **Patient positioning**, defined as mal-positioning of the patient in relation to the x-ray field (* = Optimal; - = Sub-optimal; 0 = Unacceptable)


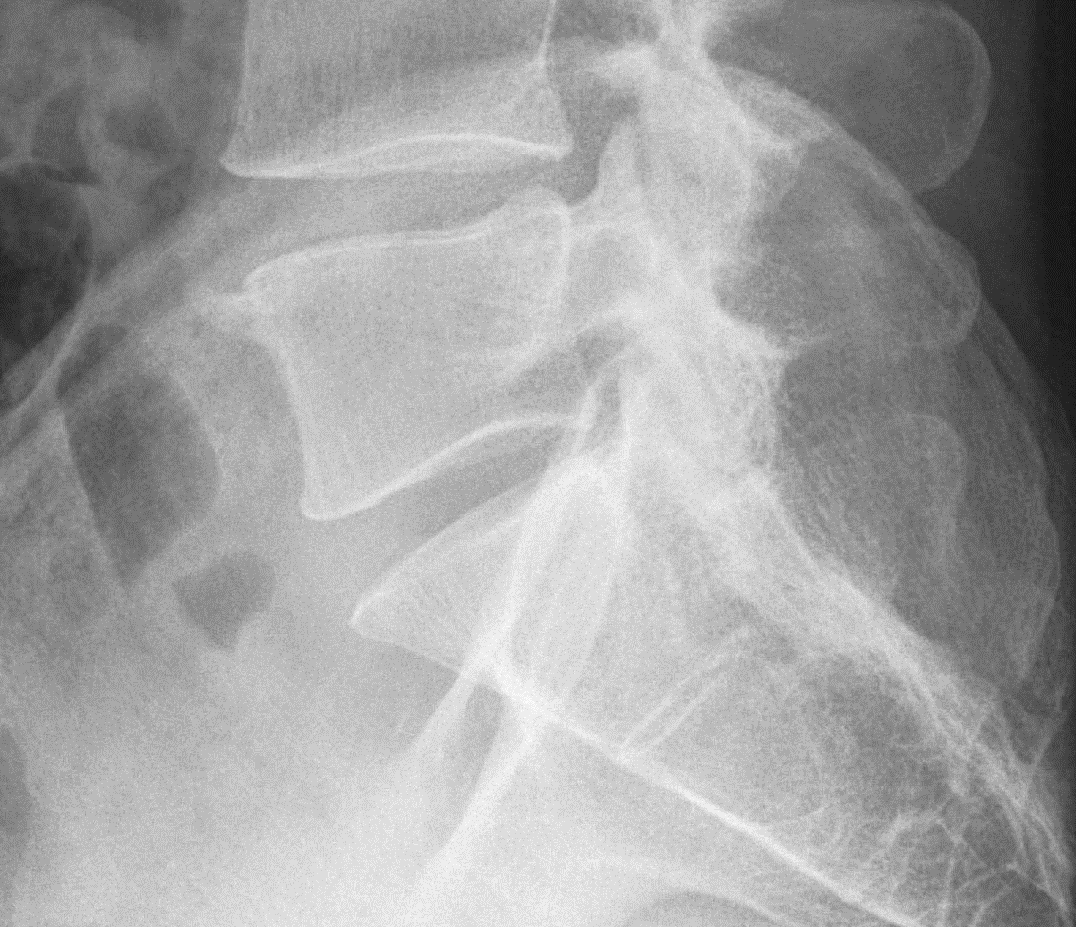


**3.1.3. Anterior border of S1**

**3.1.2. Visualization of the spinous process of L5**

**3.1.1. Linear reproduction of inf. endplate of L5 and sup. endplate of S1**

**3.1.4. Visualization of S1 and S2**
